# Psychoeducational groups versus waitlist in treatment of attention-deficit hyperactivity/impulsivity disorder (ADHD) in adults: a protocol for a pilot randomized waitlist-controlled multicenter trial

**DOI:** 10.1186/s40814-019-0401-1

**Published:** 2019-01-23

**Authors:** J. R. Vaag, M. L. Lara-Cabrera, O. Hjemdal, B. Gjervan, T. Torgersen

**Affiliations:** 1 Faculty of Nursing and Health Science, Nord University, Levanger, Norway; 2 Department of Psychology, Faculty of Social and Educational Sciences, Norwegian University of Science and Technology, Trondheim, Norway; 3 Division of Mental Healthcare, St. Olavs Hospital Trust, Trondheim, Norway; 4 Department of Mental Healthcare, Nord Trøndelag Hospital Trust, Levanger, Norway

**Keywords:** Adult ADHD, Group treatment, Patient satisfaction, Patient education, Psychoeducation, Self-efficacy, Self-management behaviors, Randomized controlled trial, Quality of life, Work participation

## Abstract

**Background:**

Psychoeducation is included in the Norwegian national guidelines for treatment of adult ADHD. Despite some promising results for the treatment of other conditions and ADHD, little is known about the efficacy of such interventions. This paper presents a protocol for a pilot randomized controlled trial featuring a psychoeducational group program for patients with ADHD. The main objective of this pilot trial is to investigate adherence, feasibility, and preliminary efficacy of a ten-session psychoeducational group designed to address specific challenges faced by adults diagnosed with ADHD.

**Methods:**

This pilot study will evaluate patient satisfaction and preliminary efficacy of a psychoeducational group treatment using a randomized waitlist-controlled trial at two different outpatient clinics in mid-Norway. All participants will receive treatment as usual, concomitant with the intervention and waitlist period. Client satisfaction (CSQ 8), general self-efficacy (GSE-6), ADHD-related quality of life (AAQoL), symptoms of ADHD (SCL-9; ASRS), and work participation will be assessed at the time of recruitment prior to randomization (T0), pre-intervention (T1), post-intervention (T2), and at 10 weeks follow-up (T3). Recruitment and dropout rates along with treatment adherence will also be evaluated.

**Discussion:**

This study offers valuable insight into the preliminary efficacy of educational programs implemented in outpatient clinics. The aim of the trial is to evaluate adherence, feasibility, patient satisfaction, and the preliminary efficacy of a psychoeducational group intervention for patients with adult ADHD and provide further insight into the design and construction of a large-scale trial. The results also offer preliminary empirical evidence to inform the development of larger and more complex studies.

**Trial registration:**

NCT03337425, Registered 9 November 2017

## Background

ADHD is a common disorder [1] that continues from childhood into adulthood in 30 to 60% of cases [2, 3]. In a large multinational study, the average prevalence rate has been estimated to be 3.4% among adults [4]. ADHD is characterized by difficulties with regard to attention, hyperactivity, and impulsivity. In addition to these persistent problems, patients often struggle with psychiatric comorbidity [5–9]. The results from previous studies indicate that as many as 90% of adult patients with ADHD struggle with one or more comorbid psychiatric conditions [10]. Of these, the most common comorbid conditions are anxiety disorders, affective disorders, substance abuse, antisocial personality disorders, and developmental disorders, such as autism and learning difficulties. ADHD among adults is also associated with impaired function in areas that include psychosocial functioning, education, work [11, 12], and executive function [1, 13]. There are minimal differences between genders in terms of symptoms, comorbidities, and function in adults with ADHD [8, 14, 15].

Previous studies [16, 17] and the “Consensus of the European Network of Adult ADHD” [18] have pointed out the importance of psychoeducation as an intervention in the treatment of adults with ADHD. These recommendations suggest that psychoeducation could offer understanding of previous difficulties and improve general functioning. Psychoeducation is not directly process-oriented, but it is effective as a therapeutic intervention for bipolar disorder [19, 20]. The results indicate higher levels of treatment compliance, better psychosocial functioning, and improved overall outcomes. Similar results have also been reported among patients with psychosis: a documented reduction of readmissions to hospital, increased compliance, and improved quality of life [21, 22].

We searched PubMed and PsycINFO and found only one randomized controlled pilot study of psychoeducation for ADHD among adults. In this study [23], a series of 11 psychoeducational sessions related to clear ADHD-specific challenges were compared with cognitive behavioral group therapy. The results indicated that both types of treatments reduced symptom severity and the negative effects of symptoms, diminished hyperactivity and impulsiveness, and improved attention and self-esteem. Patients also reported a decrease in anxiety and depression symptoms; 93.8% of patients completed the intervention. We also found a non-controlled, pilot study of psychoeducational groups for adults with ADHD and their significant others based on elements of CBT, neuropsychology, and results from interdisciplinary ADHD research. The intervention was conducted at three different clinics in Sweden and comprised 41 patients and 40 significant others. The results suggested there is an increase in knowledge of ADHD, quality of social relations, and psychological well-being after treatment [24].

Even though the results of these studies are promising, more controlled trials are needed to assess the efficacy of psychoeducation for adults with ADHD. Thus, there is a necessity to evaluate patient satisfaction and the short-term effects of psychoeducational group interventions in clinical settings. This study protocol describes an innovative education group intervention where health personnel and representatives from the ADHD user organization educate adults with ADHD to improve self-management. User involvement will occur during the intervention delivery phase. Representatives from user organizations will be peer educators co-leading two sessions. The intervention will be delivered at two centers, of which the center that has developed the intervention has limited experience with the research procedures needed to conduct a proper trial. The other center has no previous experience delivering the intervention. In order to plan a definitive trial, we must investigate the feasibility of both the research and intervention procedures, as well as evaluate patient satisfaction within the context of the intervention. We require information on how many patients will complete the intervention with evaluable data, as well as insight into the feasibility of randomization procedures and estimates of parameters required for sample calculation. This intervention is based on active participation and exchange of experiences between the participants. Measuring patient satisfaction is also an important part of fine-tuning the intervention before a large-scale trial. Hence, the purpose of our study was to investigate the feasibility, preliminary effects, and patient satisfaction with a group-based psychoeducational program consisting of ten sessions delivered to adults with ADHD using a prospective, randomized, and controlled study design.

## Methods

### Study design

This protocol describes a multicenter trial to study patient satisfaction and the preliminary efficacy of a group-based psychoeducational program for adults with ADHD and is available online at ClinicalTrials.gov (NCT03337425). The study will be reported in accordance with the Template for Interventions Description and Replication and the Consolidated Standards of Reporting Trials.

**Fig. 1:**
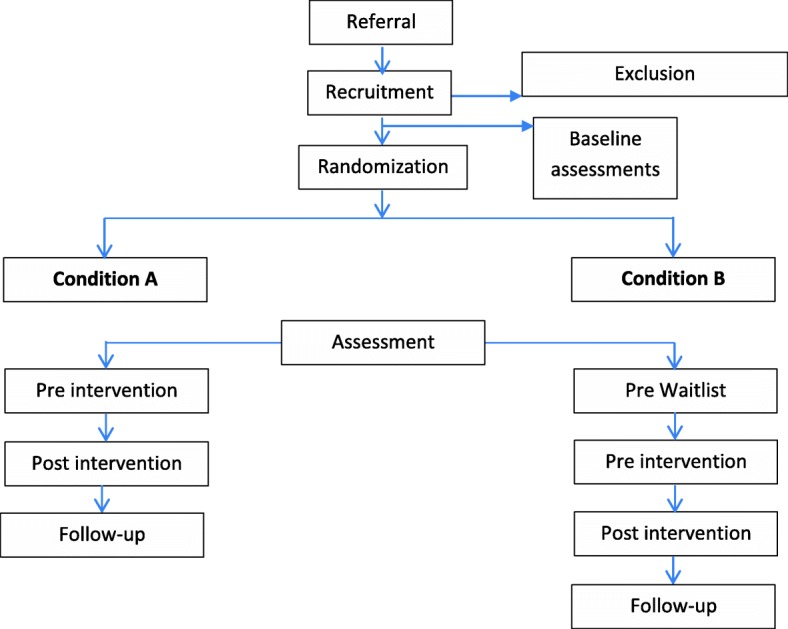
Recruitment procedure and assignment to conditions

Patients from two different outpatient clinics (Nord-Trøndelag Hospital Trust and Tiller District Psychiatric Centre) will be included. The treatment is available for patients in the Nord-Trøndelag Hospital Trust for 4 years, but it has not been evaluated nor has it been implemented at the other center. Therefore, a pilot study is warranted. All patients included in the study will receive treatment as usual concomitant to psychoeducation or waitlist. Patients will be recruited and randomly assigned to condition A, psychoeducation + treatment as usual, or condition B, waiting list + treatment as usual. There is a need for at least 30 subjects in each cohort. Eligible outpatients diagnosed with ADHD at one of the outpatient clinics could be enrolled. The inclusion and exclusion criteria are listed in Table 1, and the recruitment procedure and assignment to conditions are found in Fig. 1.

**Table 1:** Inclusion and exclusion criteria

Inclusion criteria	Exclusion criteria
Age between 18 and 67	Unable to give informed consent
Speaking a Scandinavian language	Psychosis
Confirmed ADHD diagnosis	Severe learning difficulties

#### Condition A: Psychoeducation concomitant with treatment as usual

Both conditions A and B received treatment as usual (see description under the “Condition B: Waiting list concomitant with treatment as usual” section) in addition to psychoeducation or waitlist. The group-based psychoeducational program consists of ten sessions that were ran over ten consecutive weeks. Each session consists of a lecture from a recruited expert on the topic of the session (20 min) with a following discussion of the topic (45 min) facilitated by the course leader (Table 2). All sessions are organized and led by the course leader, which also includes structured time, discussion, and closing of the session.

**Table 2:** Topic for sessions

Topic and main focus for session	Lecturer (in addition to course leader)
Introduction and presentation of the intervention Myths and facts about ADHD	Given by the course leader (nurse) and expert in ADHD (social worker or psychologist)
What is ADHD?	Nurse or social worker or psychologist with ADHD expertise
Inattention	Nurse or social worker or psychologist with ADHD expertise
Impulsivity	Nurse or social worker or psychologist with ADHD expertise
Hyperactivity	Nurse or social worker or psychologist with ADHD expertise.
ADHD and comorbidity	Medical doctor, psychologist, or experienced psychiatric nurse
Use of medications	Medical doctor specialized in ADHD
Economy	Social worker
Work and welfare	Representative from the public welfare agency Representative from ADHD Norway
Summary and closing session	Led by the course leader

#### Condition B: Waiting list concomitant with treatment as usual

Treatment as usual consists of treatment with ADHD medication for most patients. Additionally, certain patients would normally receive counseling or psychotherapy, although this is not systematically provided to every patient; thus, the randomization procedures are necessary to reduce this as a possible bias. If needed, patients are also offered assistance with regard to economy, housing, education, and work as well as contact with family and their network. The participants allocated to the waiting list control group were told to live as normal and continue with treatment.

### Data and measurement

**Fig. 2:**
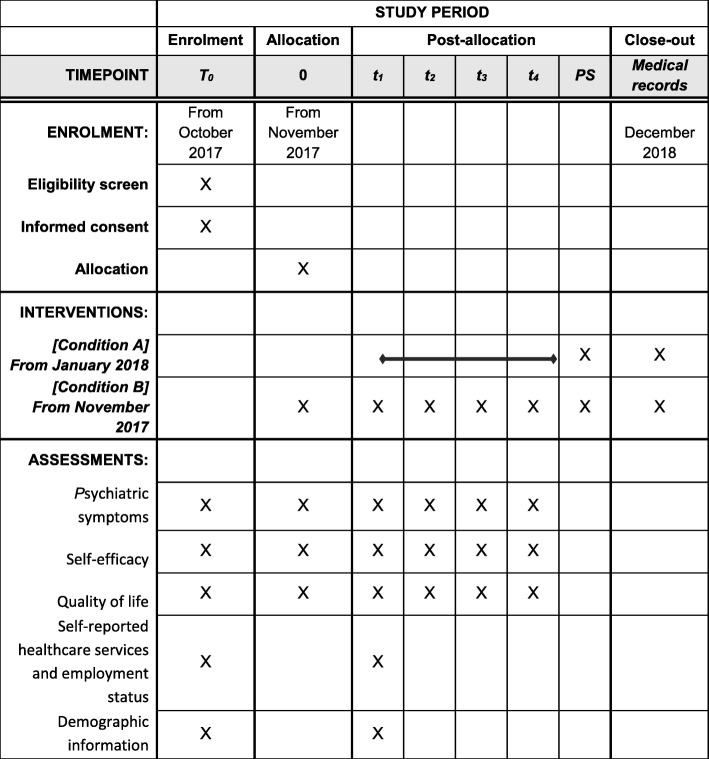
Content for the schedule of enrolment, interventions, and assessments

Clinical symptoms and function will be measured at the time of recruitment prior to randomization (T0), pre-intervention (T1), post-intervention (T2) as well as at 10 weeks follow-up (T3). The waitlist-control group will have two additional measurements during the pre- and post-waiting period. Measuring patient satisfaction and change in symptom load, general self-efficacy, quality of life, and function can serve as indicators of the preliminary efficacy of the intervention. The assessments and measurements are listed in Fig. 2.

### Fidelity scale

Fidelity will be assessed with a fidelity scale. The scale evaluates different components of intervention fidelity, such as process fidelity, content fidelity, and quality of interaction. The scale will be completed at ten time points during the intervention delivery phase.

### Medical records

Medical records will be used to collect demographic information, history of medication, as along with information about psychiatric comorbidity. Allowance to use such data is included in the participants’ informed consent.

### Self-reported demographics and questionnaires

Demographic information about age, sex, education, use of healthcare services, and employment status is collected at baseline and T1 with a self-report questionnaire.

#### Client Satisfaction Questionnaire (CSQ-8)

A modified version of the Client Satisfaction Questionnaire (CSQ-8) [25] is included in order to assess satisfaction in relation to the group-based psychoeducational program. The scale consists of eight items measured on a scale from 1 to 4; three of the items are reverse scored. A sum score between 8 and 32 indicates the level of satisfaction with the services provided. The modification of the questionnaire is an additional open question, where the participants may comment on the content and suggest changes to the psychoeducational program.

#### General Self-Efficacy Scale 6-Item (GSE-6)

The GSE-6 is a short-form version of the GSE scale [26]. It will be employed to measure general self-efficacy [27]. Self-efficacy is regarded as a protective factor in adapting to stress and chronic illness. It refers to the belief that one would be able to control and adapt to challenging demands in the future. The scale consists of six items measured on a 4-point scale from “not at all true” (1) to “exactly true” (4). The possible score range is 6–24 with a high score reflecting a higher level of self-efficacy.

#### Adult ADHD Self-Report Scale Full Edition (ASRS)

The ASRS is the World Health Organizations self-reporting scale for ADHD in adults [28]. The scale is designed to examine the current ADHD symptomatology and consists of 18 items based on the DSM-V diagnostic criteria for ADHD. The scale is divided into two parts, and each has a sum score. Questions 1–9 (part A) reflect symptoms of inattention (I), and questions 10–18 (part B) reflect symptoms of impulsivity and hyperactivity (HI).

#### Hopkin’s Symptom Checklist (SCL-90) 9-item

The SCL-90 is a self-report scale for measuring psychiatric symptoms. An ADHD-specific scale consisting of nine items from the original 90 (SCL-9) has been tested and validated for screening. This shows acceptable sensitivity with regard to measuring ADHD symptoms and is a supplement to ASRS. The SCL-9 covers the specific characteristic traits of ADHD with high scores on SCL-9 indicating ADHD-related symptom burden [29].

#### AAQoL—health-related quality of life

The AAQoL is a measure of health-related quality of life (HRQoL) that investigates quality of life and function among adults with ADHD. The AAQoL consists of 29 questions measuring HRQoL over the previous 2 weeks. The original version of the AAQoL has exhibited satisfactory psychometric properties in previous studies [30–32]. The Norwegian version has previously been validated by our research group and was found to fit well with the original version [33].

### Statistics

Statistical analysis will be conducted using SPSS [34] and STATA [35]. In order to calculate preliminary effects, chi-square statistics and *t* tests will be used for descriptive analyses of categorical and continuous variables, respectively, in addition to analysis of variance (ANOVA) for comparison of results for the control and experimental groups. Possible other predictors for the preliminary efficacy of psychoeducation will be examined using linear regression on the total sample (pre-post intervention). Intervention adherence will be calculated by assessing the response rate of participants receiving the intervention, response rate of returned questionnaires, and the number of sessions completed by intervention group participants. Recruitment and dropout rates will be evaluated using absolute and percentage frequencies.

### Sample size

This project is intended to evaluate the feasibility of implementing an educational group intervention led in co-operation with health personnel and peer educators to inform an eventual larger-scale randomized study that will allow for a more rigorous assessment of the impact of the intervention. Hence, our sample size is driven primarily by practical issues related to the number of patients needed to enroll at each site, opinions about the questionnaires, and satisfaction with the intervention. Consequently, the anticipated sample will be 60 patients—30 patients in each arm. The chosen sample size is also in accordance with the median number of participants observed in an audit of pilot and feasibility trials in the UK [36].

### Ethics

The project is registered and approved by the Regional Committees for Medical Research Ethics (REC 2016/1885) and will be conducted in accordance with the ethical principles of the Declaration of Helsinki (2013).

### User involvement

User involvement will be by advisory participation by one peer educator from the ADHD user organization with experience cooperating with mental health services. The advisory participation will occur during protocol preparation to ensure that all relevant outcomes are included. User involvement will also be determined during the intervention delivery phase. Peer educators will be co-led via two sessions.

## Discussion

This study protocol presents a pilot randomized, waitlist-controlled, multicenter trial of a psychoeducational group intervention for patients with ADHD. ADHD is a disorder with high prevalence rates associated with impaired psychosocial functioning, education, and work. Patients may therefore require education and support to cope with these conditions during everyday life. However, the effectiveness of such programs implemented in outpatient clinics remains unclear. This pilot study will assess the preliminary efficacy of the intervention on ADHD-related symptoms (ASRS and SCL-9), ADHD-related quality of life, function, and employment status as well as patient satisfaction with intervention content. If the program is beneficial for the patients and feasible for the clinics, then it may be implemented in other clinics. The results would also add to the preliminary empirical evidence needed to inform the development of larger and more complex studies.

### Main study and decision to proceed

The pilot study utilizes the same design as the intended main study to make a proper decision on whether to proceed or not. The decision to proceed will be based on the measurement of client satisfaction as well as the preliminary efficacy of ADHD-related symptoms and quality of life. With this, our study may influence the clinical practice and short-term results to empower patients in the management of their own symptoms and health treatment. The short-time impact of the intervention on function is unclear. If the decision is to proceed, then the long-term effects of the intervention will be assessed in a new main study employing the same design but with an additional 3 or 6 months of follow-up.

### Methodological strengths

To the best of our knowledge, this will be the first pilot randomized, waitlist-controlled, multicenter trial of a psychoeducational intervention for patients with adult ADHD. All participants will be recruited from outpatient clinics. These are patients with a confirmed diagnosis. The treatment is delivered in outpatient clinics and will therefore increase the ecological validity of the study within a Norwegian health care context. The outcome measurements will be based on validated instruments and well-tested in international research as well as in clinical practice. This offers useful data both for research and the clinic. Translated instruments are validated and published in international peer-reviewed journals. We used a multicenter intervention and different course leaders and lecturers to reduce the effect of individual therapeutic factors. This offers more reliable evidence on the specific effects of the intervention content.

### Methodological weaknesses

This study is not double-blinded nor did we use the “placebo intervention” other than the waiting list owing to ethical considerations. We do not compare our model to another form of treatment other than treatment as usual, but this is because there is a lack of a gold standard for psychoeducational interventions in adult ADHD. The waiting list group design has certain weaknesses, but considering the ethical aspects requiring that all patients receive treatment, the interventions were important (waiting lists are typical in these clinics).

## Conclusion

This study protocol presents a pilot randomized, controlled trial that will evaluate an innovative education program for patients with ADHD. It aims to enhance self-management behaviors. The results will be of interest to health care professionals working with ADHD patients. Additionally, our study will be of value to user organizations and policy makers administering educational programs with respect to the agenda of prevention and treatment of ADHD in adulthood.
